# Supplementary material for: Association between oral microbiome and seven types of cancers in East Asian population: a two-sample Mendelian randomization analysis
Source: Front Mol Biosci. 2023 Nov 21;10:1327893. doi: 10.3389/fmolb.2023.1327893 (PMC10702768; doi:10.3389/fmolb.2023.1327893)
Supplement: Supplementary file 1 [file DataSheet1.ZIP › Supplementary Materials/MR plots of saliva/saliva═╝/Prostate cancer/pheno.2663_to_prostate cancer_forest.pdf]

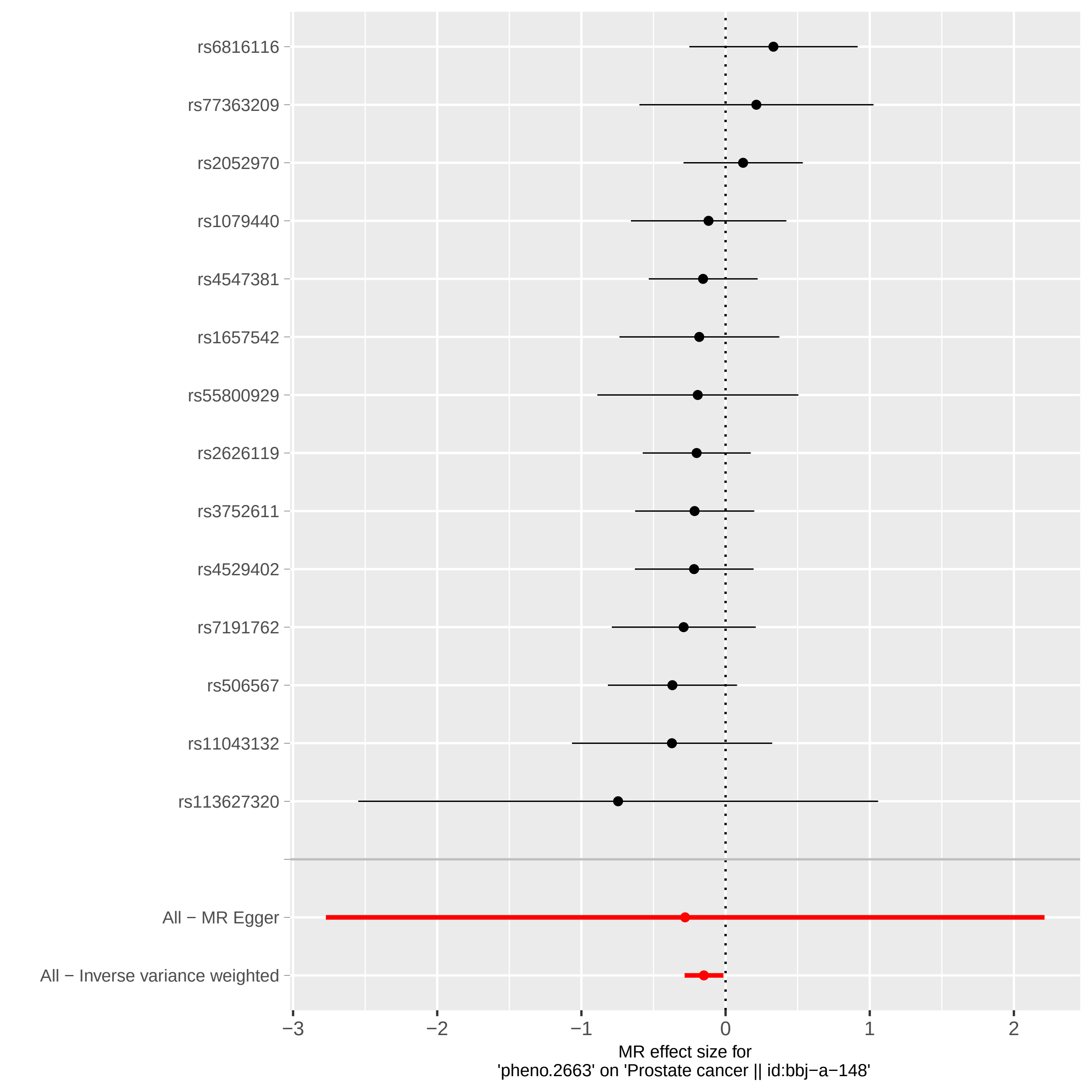
rs6816116
rs77363209
rs2052970
rs1079440
rs4547381
rs1657542
rs55800929
rs2626119
rs3752611
rs4529402
rs7191762
rs506567
rs11043132
rs113627320
All − MR Egger
All − Inverse variance weighted
−3
−2
−1
0
1
2
MR effect size for
'pheno.2663' on 'Prostate cancer || id:bbj−a−148'
